# Efficacy of cognitive behavioral therapy in children and adolescents with insomnia: a systematic review and meta-analysis

**DOI:** 10.1590/1414-431X20187070

**Published:** 2018-05-21

**Authors:** Zhong-Rui Ma, Li-Jun Shi, Ming-Hong Deng

**Affiliations:** 1 Department of Neurology, Chengdu Fifth People's Hospital, Chengdu, China; 2 Department of Hematology, Chengdu Fifth People's Hospital, Chengdu, China

**Keywords:** Insomnia, Cognitive behavioral therapy, CBT-i, Child, Adolescent, Meta-analysis

## Abstract

Insomnia is highly prevalent in children and adolescents. However, the efficacy of cognitive behavioral therapy for insomnia (CBT-i) in children and adolescents remains controversial. Therefore, this systematic review and meta-analysis aimed to assess the efficacy of CBT-i in children and adolescents. We conducted a search of PubMed, EMBASE, the Cochrane Central Register of Controlled Trials, CINAHL, and PsycINFO to select primary studies evaluating CBT-i in children and adolescents that were primarily diagnosed through standardized diagnostic criteria. The primary outcomes of the meta-analysis included sleep onset latency (SOL), wake after sleep onset (WASO), total sleep time (TST), and sleep efficiency (SE%). Six randomized controlled trials and four open-label trials met all inclusion criteria. A total of 464 participants (ranging from 5–19 years of age) were included. Based on the results from sleep logs, a significant pooled effect size was observed for SOL and SE%. However, no significant pooled effect size was found for WASO or TST. Results from actigraphy were consistent with the sleep logs. A significant pooled effect size was observed for SOL and SE%, and no significant pooled effect size was found for WASO or TST. CBT-i might be effective in the treatment of children and adolescents with insomnia.

## Introduction

Insomnia is highly prevalent in children and adolescents and has been associated with depression, anxiety, inattention problems, and poor school performance (1–6). Recent studies suggest that 5–30% of primary school-aged children, and 4–13% of adolescents experience symptoms of insomnia (3 [Bibr B04] [Bibr B05],^7^–9). The results from a large population-based longitudinal study indicate that early sleep problems predict later development of emotional and behavioral issues (10). Chronic insomnia might not only result in deleterious effects on cognitive development, mood regulation, and attention, but also produce negative effects on school performance (6,11).

The Diagnostic and Statistical Manual of Mental Disorders (DSM) and the International Classification of Sleep Disorders (ICSD) have clear definitions of insomnia. Briefly, individuals are considered to have insomnia when they display clinically relevant symptoms, which include difficulties in falling asleep and staying asleep, or not feeling rested after getting up, and presence of these problems for at least 3 months and three times per week with clinically significant consequences on daily life (12,13). In the newest ICSD-III, behavioral insomnia during childhood is included in the chronic insomnia disorder diagnosis, and the specific aspects of children are discussed within the text, such as limit-setting and sleep-onset association issues (13). Therapeutic approaches for primary insomnia involve sleep hygiene routines, psychotherapy, and pharmacological treatment. The treatment of insomnia should start with a detailed assessment of sleep latency, number of awakenings, wake after sleep onset, frequency and severity of the complaint(s), nighttime distress, and responses to related daytime symptoms (14). For children and adolescents, parents should also be educated about sleep hygiene and adequate sleep routines by their pediatricians during routine visits (15). The pharmacological treatment of insomnia in children and adolescents is a challenging problem since there is a paucity of Food and Drug Administration (FDA) approved drugs (16). As a result, non-pharmacologic treatment options for insomnia in children and adolescents have become more important. The effectiveness of various modalities of cognitive behavioral therapy (CBT) have been evaluated in adults with insomnia, including traditional face-to-face therapy, group therapy, telephone-delivered cognitive behavioral therapy, and internet-delivered or mailed self-help (17–19). Based on numerous randomized controlled trials (RCTs), CBT-i has been shown to be highly effective for both short-term and long-term outcomes in adult insomniacs (20–22). A recent meta-analysis has also shown that CBT-i is an effective treatment for adults with chronic insomnia, with clinically meaningful effect sizes (23).

Unfortunately, there have been few studies on the use of CBT-i in children and adolescents. In recent years, although several open-label trials and RCTs of CBT-i have been performed (24–26), no meta-analysis has yet assessed its efficacy. Therefore, in this study, we conducted a systematic review and meta-analysis to evaluate the efficacy of CBT-i in children and adolescents with insomnia.

## Material and Methods

### Search strategy

This study followed the methodological and reporting guidelines from the Cochrane Handbook for Systematic Reviews of Interventions (27). We searched five electronic databases: PubMed, EMBASE, Cochrane Central Register of Controlled Trials (CENTRAL), CINAHL, and PsycINFO from inception to November 2017. The search strategy has been detailed in Table 1. No language restrictions were imposed. Additionally, reference lists of relevant reviews were used to find eligible trials.

**Table 1.** Search strategy.

Categories	Search terms
Participants	(child OR children OR adolescent* OR pediatric* OR paediatric*) AND
Disease	(sleeplessness OR ‘chronic insomnia’ OR insomniac OR insomnia OR insomni* OR ‘sleep initiation and maintenance disorders’) AND
Interventions	(CBT OR ‘cognitive behavioural therapy’ OR ‘cognitive behavioral therapy’ OR ‘cognitive behavior therapy’ OR ‘cognitive behavioural therapies’ OR ‘cognitive behavioral therapies’ OR ‘cognitive behavior therapies’ OR ‘cognitive analytic therapy’ OR ‘sleep hygiene’ OR ‘stimulus control’ OR ‘relaxation’ OR ‘relaxation therapy’ OR ‘relaxation techniques’ OR ‘behavior modification’ OR ‘behavior therapy’ OR ‘cognitive therapy’ OR ‘imagery’ OR ‘biofeedback’ OR ‘counseling’ OR ‘family therapy’ OR ‘psychoanalytic therapy’ OR ‘psychotherapy’)

### Eligibility criteria

We applied the following inclusion criteria: i) primary prospective clinical studies that ii) evaluated CBT-i in children and adolescents (aged less than 20 years) with iii) a primary diagnosis of insomnia according to the standardized diagnostic criteria from the fourth or fifth editions of the Diagnostic and Statistical Manual for Mental Disorders (DSM-IV, DSM-V) (12,28), and the second or third editions of the International Classification of Sleep Disorders (ICSD-II, ICSD-III) (29,13). The interventions could include multimodal therapies, including sleep education, stimulus control, sleep restriction, relaxation, sleep hygiene, and cognitive techniques. We excluded trials conducted with adults (aged 20 years and older) or those studies including patients with comorbid medical or psychiatric disorders (e.g., depression, anxiety, epilepsy or pain).

### Outcome measures

The primary outcomes of interest included sleep onset latency (SOL), wake after sleep onset (WASO), total sleep time (TST), and sleep efficiency (SE%). Outcomes from questionnaires and actigraphy were also included in the analysis. When reported, we collected both the post-treatment outcome and the follow-up outcome.

### Data extraction and quality assessment

Two authors (LJS and MHD) independently screened the titles and abstracts according to the eligibility criteria to select relevant studies. Then, a standardized data extraction form, including age, gender, country, diagnosis, sample size, study design, setting, duration of follow-up, main outcomes, funding sources, and quality assessment, was used to extract the data from the selected studies. All disagreements were resolved by a third author (ZRM). Study quality was assessed using the Risk of Bias Assessment Tool from the Cochrane Handbook (27).

### Statistical Analysis

We performed a meta-analysis using Review Manager software (RevMan, the Cochrane Collaboration, Denmark). The standardized mean difference (SMD) was used as the effect size for continuous outcomes. The I^2^ statistic was used to estimate the percentage of variation across studies that resulted from heterogeneity rather than chance, and an I^2^ value of 50% or higher was taken to indicate significant statistical heterogeneity. A random-effects model was used, as there was expected heterogeneity from the different CBT modalities. The overall effect sizes were calculated based on the pooled proportions and 95% confidence intervals (CIs). We performed a sensitivity analysis that included only RCTs. Subgroup analyses were conducted with a random-effects model to determine a conceivable reason for heterogeneity.

## Results

### Search results

**Figure 1.**
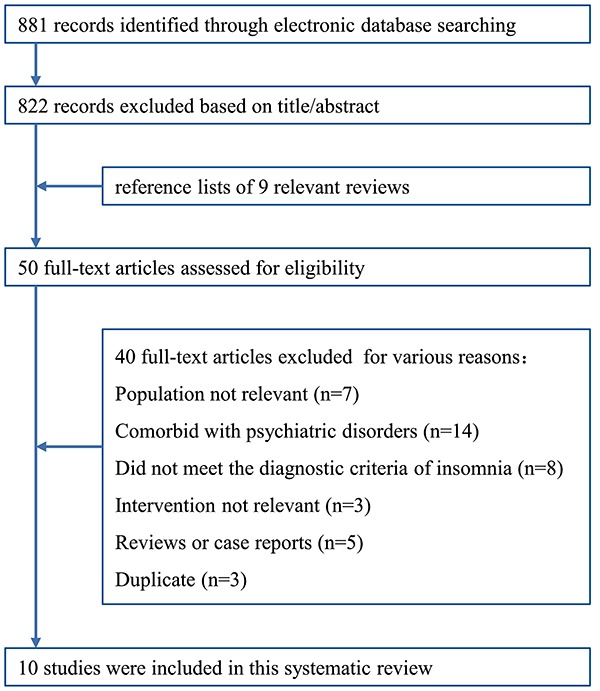
Flowchart of study selection.

The study selection process is diagrammed in Figure 1. A total of 881 records were identified and screened through the initial search strategy, and the reference lists of 9 relevant reviews were also screened. A total of 822 records were excluded based on irrelevant titles and abstracts. Then, 40 articles were excluded after a full-text review. Ten studies, consisting of six RCTs and four open-label trials, met all the eligibility criteria (24–26,30–36). Among the included studies, two (one RCT, one open-label trial) were one-year follow-up results of previous studies (35,36).

### Characteristics of included studies

The characteristics of the ten included studies are presented in Supplementary Table S1. The ten included studies were published from 2010–2017 and included 464 participants, of which 167 were male and 297 were female. The sample size per study ranged from 18 to 116 patients, and the mean age across all participants was 12.7 years (age range: 5–19). Five studies were from the Netherlands (24,31–33,36), four were from Germany (26,30,34,35), and one was from Australia (25). The definition of insomnia was based on the DSM-IV, DSM-IV-TR, DSM-V, ICSD-II, or ICSD-III with one study using the Holland Sleep Disorder Questionnaire (HSDQ). Various CBT modalities were used across the included studies, and the CBT interventions differed in terms of treatment format (i.e., individual, group, online, web-based, or telephone-based). The duration and the number of treatment sessions were consistent across ninety percent of the included studies. The six RCTs were rated as high-quality studies, while the four open-label trials were rated as low-quality studies.

### Meta-analysis of primary outcomes

**Figure 2.**
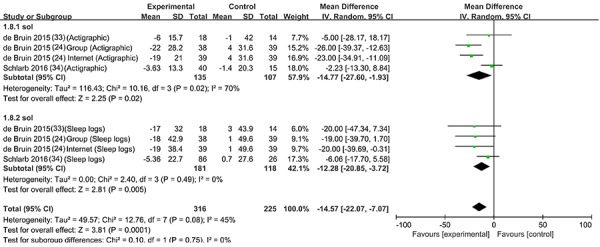
Meta-analysis of the effect of cognitive behavioral therapy for insomnia on sleep onset latency (SOL).

**Figure 3.**
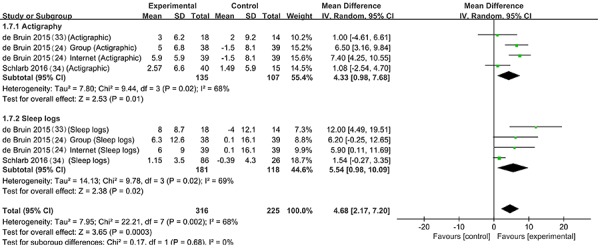
Meta-analysis of the effect of cognitive behavioral therapy for insomnia on sleep efficiency (SE%).

**Figure 4.**
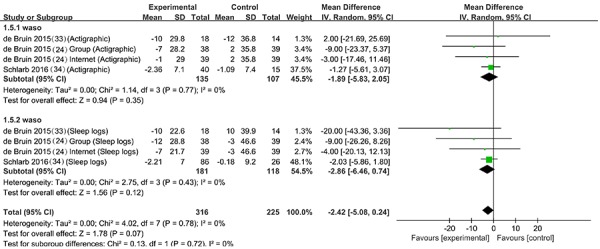
Meta-analysis of the effect of cognitive behavioral therapy for insomnia on wake after sleep onset (WASO).

**Figure 5.**
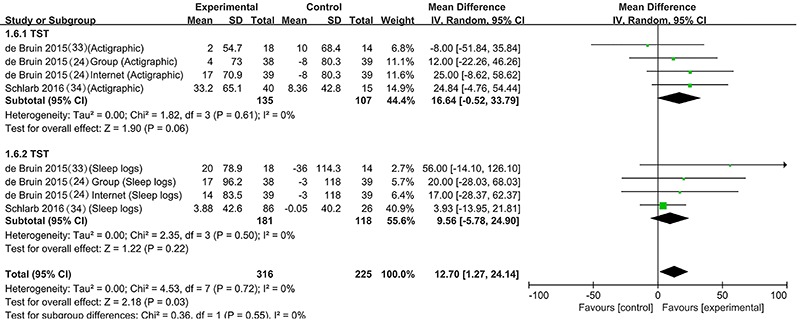
Meta-analysis of the effect of cognitive behavioral therapy for insomnia on total sleep time (TST).

Three studies reported sufficient data to assess the effects of CBT-i relative to untreated control conditions. One study compared participants treated with two treatment modalities of CBT (i.e., group therapy and guided internet therapy) against untreated control participants on a waiting list (24). Data from the sleep logs and actigraphy measures were included in the meta-analysis. Based on the results from actigraphy, a significant pooled effect size was observed for SOL (MD=-14.77, 95%CI, -27.60 to -1.93, P=0.02, I^2^=70%, Figure 2) and SE% (MD=4.33, 95%CI, 0.98 to 7.68, P=0.01, I^2^=68%, Figure 3). However, no significant pooled effect sizes were found for WASO (MD=-1.89, 95%CI, -5.83 to 2.05, P=0.35, I^2^=0%, Figure 4) or TST (MD=16.64, 95%CI, -0.52 to 33.79, P=0.06, I^2^=0%, Figure 5). Results from sleep logs were consistent with the actigraphy. A significant pooled effect size was observed for SOL (MD=-12.28, 95%CI, -20.85 to -3.72, P=0.005, I^2^=45%, Figure 2) and SE% (MD=5.54, 95%CI, 0.98 to 10.09, P=0.0003, I^2^=68%, Figure 3), and no significant pooled effect sizes were found for WASO (MD=-2.86, 95%CI, -6.46 to 0.74, P=0.12, I^2^=0%, Figure 4) or TST (MD=9.56, 95%CI, -5.78 to 24.90, P=0.22, I^2^=0%, Figure 5). The SOL and SE% results indicated that CBT markedly shortened the average time to enter sleep after lights-out and improved sleep efficiency compared to control. Besides, there was significant heterogeneity observed in the SOL and SE% analyses of actigraphy, while heterogeneity was minimal in the other analyses.

### Systematic review of included studies

#### Review of included RCTs

The two RCTs by de Bruin et al. (24,33), employed the Chronic Sleep Reduction Questionnaire (CSRQ) and the insomnia scale from the HSDQi. The CSRQ was used to measure the symptoms of chronic sleep reduction and the HSDQi was used to diagnose common sleep disorders; higher scores of these two scales indicated greater chronic sleep reduction. Results from the CSRQ and HSDQi showed significant decreases in total scores after six weeks of CBT-i or untreated waiting list, with CBT-i treated participants displaying more significant decreases in total scores compared to the untreated waiting list controls. CBT-i (whether through group therapy or guided internet therapy) displayed positive effects, and the improvements in HSDQi and CSRQ scores were maintained at follow-up. Compared with the guided internet therapy, the group therapy showed a slightly smaller increase between baseline and post-test SE based on actigraphy, and a slightly larger increase of SE from post-test to follow-up based on sleep logs. However, none of these differences were significant. In a recent study, de Bruin et al. also assessed the psychopathology after insomnia treatment at 6 and 12 months follow-up (36). The results of this study were based on his previous RCT. The follow-up data demonstrated that internet and face-to-face CBT for insomnia treatment achieves long-term reduction in adolescent psychopathology.

Paine and Gradisar (25) conducted a RCT to evaluate CBT-i in school-aged children with insomnia that revealed significant improvements in SOL, WASO, and SE% post-CBT, based on both a sleep/wake diary and actigraphy, effects which were maintained during the follow-up period. Moreover, the authors considered that the improvements in insomnia symptoms would be associated with decreases in symptoms of anxiety and depression. Thus, the Spence Children's Anxiety Scale (SCAS) and the Short Mood and Feelings Questionnaire (SMFQ) were used to assess the symptoms of anxiety and depression, and the Pediatric Daytime Sleepiness Scale (PDSS) was used to assess daytime sleepiness. Higher scores in these three scales indicate greater levels of anxiety symptoms, depressive symptoms, and sleepiness. Results from the SCAS, SMFQ, and PDSS indicated that CBT-i was effective in anxiety symptoms but not in depression symptoms or daytime sleepiness.

Schlarb et al. (30) evaluated the effect of a six-session multicomponent CBT-i on children with insomnia. The components included stimulus control, sleep hygiene education, and cognitive therapy. The sleep symptoms were assessed by the Children Sleep Habit Questionnaire (CSHQ) and the Sleep Disturbance Scale for Children (SDSC). Results from both scales evaluated 1 week after treatment indicated that the CBT-i group showed significant improvement compared to the untreated waiting list controls, including bedtime resistance and sleep anxiety. In another RCT by Schlarb et al. (34), CBT-i was implemented in three sessions for children and parents, respectively. A total of 112 children with chronic childhood insomnia were randomly assigned to a wait-list or CBT-i group. After the 3-, 6-, and 12-month follow-ups, the long-term effectiveness of CBT-i in treating school-age children was demonstrated.

#### Review of Included Open-Label Studies

de Bruin et al. (32) applied CBT-i to treat 58 adolescents with insomnia, and the Adolescent Sleep Hygiene Scale (ASHS) was used to assess sleep hygiene, with higher scores indicating more adequate sleep hygiene practices. After treatment, the total ASHS score as well as the ‘physiological’, ‘cognitive’, ‘emotional’, ‘daytime sleep’, ‘bedtime routine’, and ‘cognitive-emotional’ domain scores showed significant improvements in CBT-treated participants compared to untreated waiting list controls. In another open-label study by de Bruin et al. (31), six-week sessions of CBT in a group or individual internet setting were compared in a small sample. HSDQi and CSRQ scores indicated that both group and individual internet settings produce strong and comparable alleviating effects on sleep disturbances, insomnia complaints, and symptoms of chronic sleep reduction.

In the Schlarb et al. (26) open-label study, a multimodal program termed ‘JuSt’ was used to treat insomnia in adolescents. The JuSt program consisted of psychoeducation about sleep and sleep disorders, stimulus control, sleep hygiene, cognitive therapy, hypnotherapy, and progressive muscle relaxation. After the JuSt program, the sleep diary data revealed that the SOL and SE% were all significantly improved in the treated participants. The total SDSC and Youth Self Report Rating scores showed significant improvements in initiating and maintaining sleep (26). Roeser et al. (35) 1-year follow-up study suggested that the JuSt program represented a potent intervention to sustainably reduce insomniac complaints in adolescents. All parameters were still significantly different from the baseline level at 12-month follow-up, indicating a stable improvement.

## Discussion

In this systematic review and meta-analysis, we identified seven eligible studies that assessed CBT-i on children and adolescents with insomnia. From the meta-analysis, we found that CBT-i had a significant impact upon insomnia, as demonstrated by improved SOL and SE%. However, no significant improvements were found in WASO or TST. From our systematic review of the seven eligible studies, CBT-i appears to be effective in alleviating insomnia symptoms over the full duration of clinical follow-up.

To minimize the heterogeneity, we only included the data from high-quality RCTs. Data from both sleep logs and actigraphy measures were included in our meta-analysis, and the sleep log results were generally consistent with those measured by actigraphy. The treatment duration and the number of treatment sessions were almost six, with the exception of Schlarb's RCT. However, the three studies by Schlarb et al. (26,30,34) also provided CBT-i treatment sessions for the parents. These factors may have contributed to the heterogeneity of SOL and SE%. In the study of Paine and Gradisar (25), most of the participants were children, so the completion of sleep diaries needed their parent's assistance.

Insomnia is a serious sleep disorder that produces disruptive effects on emotional and behavioral development in young patients. In this study, we found that CBT-i is effective in improving the parameters and symptoms of insomnia. Similar results have been reported in previously published CBT-i meta-analyses on adults with insomnia, which showed marked improvements in SOL, WASO, and SE%. Moreover, de Bruin et al. (32) has reported that CBT-i significantly decreases the total ASHS score, a metric which has been positively correlated with sleep duration and SE%, and negatively correlated with daytime sleepiness (37,38). In college students, CBT-treated participants show significant improvements in sleep efficiency, SOL, number of awakenings, WASO, and sleep quality compared to untreated waiting list controls (39). In addition, Dewald-Kaufmann et al. (40) has shown that self-reported sleep issues and depressive symptoms in adolescents with chronic insomnia can be improved through combining gradual sleep extension and better sleep hygiene.

This systematic review and meta-analysis has several limitations. First, due to the limited number of existing RCTs on children and adolescents with insomnia, this meta-analysis was solely based on data extracted from two RCTs. Second, the three open-label studies included in the systematic review were of low-quality. Third, the included studies displayed considerable differences in CBT modalities. Fourth, the clinical scales for assessing sleep disturbance varied across the included studies, such as SOL, TST, HSDQ, and ASHS.

This systematic review and meta-analysis provides evidence that CBT-i may be effective in the treatment of children and adolescents with insomnia. CBT-i appears to improve the SOL and SE% in these patients and may also be effective in treating anxiety symptoms. Due to the limited number of included studies, these results should be confirmed by further large-scale RCTs focused on children and adolescents with insomnia.

## Supplementary Material

Click here to view [pdf]
